# Intimate partner violence and depression among women in rural Ethiopia: a cross-sectional study

**DOI:** 10.1186/1745-0179-5-8

**Published:** 2009-04-28

**Authors:** Negussie Deyessa, Yemane Berhane, Atalay Alem, Mary Ellsberg, Maria Emmelin, Ulf Hogberg, Gunnar Kullgren

**Affiliations:** 1 School of Public Health, Addis Ababa University, PO Box 9086, Addis Ababa, Ethiopia; 2 Addis Continental Institute of Public Health, PO Box 26751/1000, Addis Ababa, Ethiopia; 3 Department of Psychiatry, Faculty of Medicine, PO Box 9086, Addis Ababa, Ethiopia; 4 International Centre for Research on Women (ICRW) 1120 20th St. N.W., Suite 500 North Washington, D.C. 20036, USA; 5 Epidemiology, Department of Public Health and Clinical Medicine, Umeå University, Umeå, Sweden; 6 Obstetrics & Gynaecology, Department of Clinical Science, Umeå University, Umeå, Sweden; 7 Psychiatry, Department of Clinical Science, Umeå University, Umeå, Sweden

## Abstract

**Background:**

Studies from high-income countries have shown intimate partner violence to be associated with depression among women. The present paper examines whether this finding can be confirmed in a very different cultural setting in rural Ethiopia.

**Method:**

A community-based cross-sectional study was undertaken in Ethiopia among 1994 currently married women. Using the Composite International Diagnostic Interview (CIDI), cases of depressive episode were identified according to the ICD-10 diagnosis. Using a standardized questionnaire, women who experienced violence by an intimate partner were identified. A multivariate analysis was conducted between the explanatory variables and depressive status of the women, after adjusting for possible confounders.

**Results:**

The 12-month prevalence of depressive episode among the women was 4.8% (95% CI, 3.9% and 5.8%), while the lifetime prevalence of any form of intimate partner violence was 72.0% (95% CI, 70.0% and 73.9%). Physical violence (OR = 2.56, 95% CI, 1.61, 4.06), childhood sexual abuse (OR = 2.00, 95% CI, 1.13, 3.56), mild emotional violence (OR = 3.19, 95% CI, 1.98, 5.14), severe emotional violence (OR = 3.90, 95% CI, 2.20, 6.93) and high spousal control of women (OR = 3.30, 95% CI, 1.58, 6.90) by their partners were independently associated with depressive episode, even after adjusting for socioeconomic factors.

**Conclusion:**

The high prevalence of intimate partner violence, a factor often obscured within general life event categories, requires attention to consider it as an independent factor for depression, and thus to find new possibilities of prevention and treatment in terms of public health strategies, interventions and service provision.

## Background

Depression is more common among adult women, with in most countries nearly twice the rate seen in men [1,2]. This gender difference has been found throughout the world using a variety of diagnostic schemes or interview methods [3-5]. In a multi-country study undertaken across 15 centres in four continents with a sample of 26,969 patients attending primary health care, the prevalence of ICD-10 current depressive episode among women and men was 12.5% and 7.1% respectively [6]. Of the few studies conducted in Africa, one study in Uganda, using the Beck Depression Inventory (BDI) with 13 items, reported a prevalence of probable clinically significant depression of 17.4% (24.5% for women and 13.9% for men) [7]. A study carried out in Ethiopia using CIDI showed lower prevalence. Another study by Awas and co-workers in 1999, among 600 adults in Butajira in Ethiopia, assessed depression using the Amharic version of CIDI. The lifetime prevalence of an ICD-10 diagnosis of depressive episode was 3% (4.5% in women and 0.6% in men) and the one-month prevalence 2.4% (3.8 in women and 0.2% in men) [8].

Clinical and epidemiological studies concur that women are at greater risk of depression. Some of the possible explanations include the hormonal hypothesis, i.e. that the rate of depression in females increases during mid-puberty and later ages [3], the role of higher rates of prior anxiety in the female population [9], and the higher chronic burden faced by women [5]. Parental genetic markers are known to influence the rate of depression among children, but there is some doubt whether this genetic vulnerability differs between men and women [10,11]. Other hypotheses for the sex difference in depression are the type of adverse life events women experience, such as those relating to daily housing problems, low educational levels and unemployment [12].

The subordination of women is also associated with a number of psychosocial stressors, one of them being violence, which has been associated with adverse mental health consequences [13,14]. Studies from different settings in the US have shown intimate partner violence to be associated with depression among women [15-17]. According to studies conducted in clinical or facility-based settings, partner violence is associated with depression and attempted suicide [18-20]. Community-based studies have also reported severe forms of partner violence to be associated with increased rates of depression [20,21]. Further, childhood sexual abuse is associated with adult-onset depression in both men and women, although the occurrence of such abuse is more common in girls than in boys [22]. A possible explanation for why violence against women generates adverse mental health consequences including depression is recurrent fear and learned helplessness [21,23].

With the exception of a Palestinian study [21], the above studies on the relationship between intimate partner violence and depression drew on population data from high income countries. Further, few community-based studies use depression scales as a diagnostic measure, or structured diagnostic interviews which allow for categorical diagnosis of depressive episodes. In addition, little is known about the relationship between violence by an intimate partner and depression in an African context, where many people live in extreme poverty, polygamous marriages are prevalent and the culture is very different from that of most high income countries.

According to the WHO multi-country study on *Health and life events among women*, the prevalence of physical and sexual violence perpetuated by an intimate partner was high, in Butajira, a rural region of Ethiopia, 48% and 58% respectively [13]. The aim of the present study is to examine the relation between intimate partner violence and depression in a community-based study in Butajira using structured diagnostic interviews.

## Methods

### Study design and Study setting

This was a community-based cross-sectional survey conducted in Ethiopia in two districts called Meskan and Mareko. The study was part of the WHO multi-country study on women's health and life events and was conducted between January and December 2002 [24,25]. The districts are about 130 km south of Addis Ababa, and have a total population of about 257,500, of which 51.1% are females, as projected from the 1994 census of Ethiopia. Women aged between 15 and 49 years make up 24.8% of the population [26]. The district is organized administratively into small units termed peasant associations in rural areas and urban dwellers' associations in semi-urban areas; both units are commonly referred to as *kebeles*. Most of the population (87%) live in rural villages in which households primarily depend on subsistence farming [26], the economic mainstay of the district, comprising more than 85% of the economy. The main staple foods are maize and *ensete *(false banana); *khat*, pepper and coffee are the main cash crops [27].

Within the area, there is a demographic surveillance site called the Butajira Rural Health Programme (BRHP). The Butajira Rural Health Programme is a Demographic Surveillance Site (DSS) that includes one semi-urban dwellers' association and nine rural peasant associations selected in 1986 from the Butajira district using the probability proportionate to size technique [27,28]. The current study was conducted within the Butajira Rural Health Programme demographic surveillance site (BRHP), utilizing its household database as the sampling frame [27].

As part of the WHO multi-country study, women living in the Butajira Rural Health Programme (BRHP) were the source population. Women fulfilling the following inclusion criteria were selected: aged between 15 and 49 years, residents of the Demographic and Surveillance Site of the BRHP for at least the past three months, and included on the list of women in the database [25]. In this study we excluded women who were not currently married.

### Sampling method

Based on the prevalence of physical violence in the study site of 45% [29], and the reported prevalence of depression among women in a previous study from the area [30], we assumed the prevalence of depression to be 7.0% among non-abused women and 10% among abused women. In order to obtain the maximum sample size at a 95% confidence level and 80–95% power certainty, to detect a lower associated odds of depression of about 1.4, a minimum of 3048 women were necessary. To compensate for possible non-response, an additional ca. 5%, or 152 women, were added. Thus, a total of 3200 women were required. We managed to recruit 3016 women between the ages of 15 and 49, a response rate of 94.25%. In this paper, data from 1,994 currently married women were used.

Since 85% of the population resides in rural areas, an equivalent percentage of the sample was drawn from rural peasant associations. Additionally, in order to ensure that the number of women was equally distributed in each clustered peasant association, we recruited participants in proportion to population size. The database of each *kebele *obtained from the BRHP provided a sampling frame that contains the women's name, a unique identity number and a household number. Women were selected by simple random sampling from the database of each *Kebele *using SPSS for Windows software. In order to find women at risk of violence by an intimate partner, all 1994 currently married women were included in the present study.

### Data Collection and Management

Thirty women who had completed high school education and had experience of data collection were trained for a total of three months in the Composite International Diagnostic Interview (CIDI), version 2.1 [31], and a WHO standardized questionnaire of life events and health [25]. Data were collected after conducting a pilot test in villages outside the survey area.

### Measurements

Cases of depressive episode were identified by interview using (Section E of) the Amharic version of the Composite International Diagnostic Interview (CIDI), version 2.1, which was validated in Addis Ababa [32]. In this study, to ascertain cases of depressive episode we used F32 of the International Classification of Diseases, 10^th ^edition (ICD-10) [33], which is equivalent to major depression in the Diagnostic and Statistical Manual (DSM-IV) [34] of mental disorders.

Intimate partner violence and socioeconomic characteristics of the women were collected by means of the WHO standardized questionnaire [24]. The questionnaire included questions relevant for measuring physical, sexual and emotional violence by an intimate partner and sociodemographic characteristics. Labelling of various forms of intimate partner violence, including physical, sexual and emotional violence, is shown in Table 1. Women who had experienced physical violence were further asked whether it occurred during their last pregnancy, whether any of the physical violence resulted in physical injury, and whether the violence began more than 12 months prior to the interview. Spousal control over the respondent was measured and categorized using six items referring to what a woman was able to do without permission from her spouse (Table 1).

**Table 1:** Measurement and labelling criteria of violence by an intimate partner among currently married women in Butajira, rural Ethiopia

Measurement	Labelling criteria
**Physical violence**	

*Moderate*	Experiencing any of the six items is considered as experiencing ***'****physical violence'*
• Being slapped or **object **thrown at person	
• Being pushed or shoved	
***Severe***	
• Being hit with a fist or object	Experiencing only one or both of the first two items is labelled '*moderate*'; if any of the other four items were experienced, this was labelled '*severe*' physical violence
• Being kicked, dragged or beaten	
• Being choked or burned intentionally	
• Threatened with or use of weapon/knife	

**Sexual violence**	

• Physically forced **to have **sex	Experiencing any of the three items **is labelled as ' *sexual ****violence'*
• Forced **to have **sex that made her afraid	
• **Presence **of degrading sex	

**Emotional violence**	

• **Being belittled **or humiliated in front of others	Experiencing any of the three items is **considered *'emotional violence'***
• **Subjected to fear or intimidation**	Experiencing only **one **item **is labelled *'moderate'*; experiencing **two or **more **items **is labelled *'severe' ***emotional violence
• **Threatened, either you **or someone **close**	

Any form of intimate partner violence	Experiencing **any physical**, sexual or emotional violence is considered as experiencing ***'intimate ****partner violence'*

The Amharic translated version of questions with ambiguous terminologies used to measure intimate partner violence was discussed in two series of focus group discussions to ensure local understanding.

Sexual violence during childhood was measured anonymously using a picture showing a happy and a sad woman. The interviewees were instructed to mark the picture of the "sad woman" representing being sexually violated as a child before age 15 years, and to mark on the picture of the "happy woman" representing a woman who had not been sexually violated as a child. The enumerators were kept blind to the marks, and the marked pictures were placed in an envelope sealed by the interviewee.

To control for confounding effects and to assess any independent effect of intimate partner violence on depression, socioeconomic and sociocultural characteristics were included. Age was grouped into four categories; residency was grouped into urban and rural; and educational status was grouped as 'no formal education', 'elementary' for grades 1 to 6, and 'high school and above' for education longer than 6 years. Religion was categorized as Christian or Muslim, and occupational status was categorized into four groups: no job, selling/trading, seasonal work (working in any available unskilled job), and other. Poverty status of women was categorized into three groups, and khat chewing was categorized by frequency of use, as described elsewhere [35]. In addition, marriage type was categorized as polygamous or monogamous. As a predominantly Muslim community, most women did not respond to the question on alcohol use, so it was omitted from the analysis.

### Data Analysis

The sociodemographic characteristics and experience of intimate partner violence of the participants were summarized using descriptive statistics. In summarizing the data, totals were indicated when figures did not exactly sum 1994. The 12-month status of association between intimate partner violence and depressive episode in the past 12 months was explored using crude and adjusted odds ratios and their 95% confidence intervals. To examine the presence of any association between various types of intimate partner violence and depressive episode, we used multiple regression. In the multiple regression, socioeconomic characteristics that were statistically associated with depressive episode or intimate partner violence were included to control for possible confounding effects. We set the criteria for entry and retention in the regression equation ∝ at 0.05.

### Ethical Considerations

The study was approved by the ethics and publication committees of the Medical Faculty of Addis Ababa University, the Ethiopian Science and Technology Commission and Umeå University in Sweden. As part of the multi-country study on violence against women, the current study followed WHO ethical and safety guidelines [24]. Informed consent was obtained from each woman participating in the study and from the local formal and informal leaders. Privacy and confidentiality were also maintained throughout the data collection. During the data collection period and for one year after data collection, a counselling service was instituted in the area for those in need.

## Results

### Sample

Of the 1994 currently married women age, 44% were in the 25–34 year age group and 33% in the 35–44 year age group. The mean age was 31.6 (SD, ± 7.8) years. Eighty-five percent of the women were illiterate, 73% were Muslim, 87% were from a rural community, and 31.3% were living in a polygamous marriage (Table 2). The sociodemographic characteristics of the study subjects did not differ with the parameter, the demographic surveillance database, in age distribution, ethnicity, religion, type of marriage or level of education. The only difference observed was residency, in that urban women were underrepresented in the current study (15% in the study versus 24% in the database). Comparison of difference in 12-month prevalence of depressive episode by sociodemographic characteristics was computed crudely, and depressive episode was associated to a significant extent with residence, khat chewing, occupation and poverty status of women. Similarly, lifetime experience of any form of intimate partner violence was computed by sociodemographic characteristics crudely: experiencing any form of intimate partner violence was associated significantly with age group, lower educational status, khat chewing, and the woman's occupational status (Table 2).

**Table 2:** Sociodemographic characteristics and association with 12-month depressive episode and lifetime prevalence of any form of intimate partner violence of currently married women in Butajira, rural Ethiopia (n = 1994)

Characteristics	Sample	Depressed No. (%)	P-value	Intimate partner Violence No. (%)	P-value
Age group (years)					
15 – 24	370	15 (4.1)	0.570	241 (65.1)	0.001
25 – 34	875	40 (4.6)		676 (77.3)	
35 – 44	667	35 (5.2)		460 (69.0)	
45 – 49	82	6 (7.3)		58 (70.8)	

Residency					
Urban	254	5 (2.0)	0.023	181 (71.3)	0.789
Rural	1740	91 (5.2)		1254 (486)	

**Education**					
No formal education	1699	82 (4.8)	0.980	1226 (72.2)	0.012
Elementary	248	12 (4.8)		184 (74.2)	
High school & above	47	2 (4.3)		25 (53.2)	

Religion					
Christian	544	21 (3.9)	0.220	380 (69.9)	0.198
Muslim	1450	75 (5.2)		1055 (72.8)	

Type of marriage					
Polygamous	624	29 (4.6)	0.814	466 (74.7)	0.069
Monogamous	1370	67 (4.9)		969 (70.7)	

Khat chewing					
Never	741	28 (3.8)	0.017	478 (64.5)	0.001
Less than once/month	302	9 (3.0)		237 (78.5)	
At least once/week	951	59 (6.2)		720 (75.7)	

Occupation (n = 1993)					
No job	1275	53 (4.2)	0.040	907 (71.1)	0.04
Selling/trading	611	34 (5.6)		449 (73.5)	
Seasonal work	71	7 (9.9)		53 (74.6)	
Other	36	2 (5.6)		25 (69.4)	

Poverty status					
Very poor	291	18 (6.2)	0.080	210 (72.2)	0.317
Moderate	1245	62 (5.0)		908 (72.9)	
Relatively better off	458	16 (3.5)		317 (69.2)	

### Prevalence

In this study, the overall 12-month prevalence of depressive episode was 4.8% (95% CI, 3.9% and 5.8%), and the lifetime prevalence of any form of intimate partner violence was 72.0% (95% CI, 70.0% and 73.9%). Lifetime prevalence of physical violence by an intimate partner was 49.5% (95% CI, 47.4% and 51.7%). More than a quarter of the women had experienced moderate or severe forms of emotional violence, and more than half were partially or completely restricted in what they could do, requiring permission from their spouse. Almost one in 10 women reported experience of sexual violence before age 15 (Table 3).

**Table 3:** Prevalence and 95% confidence intervals of depressive episode and intimate partner violence among currently married women in Butajira, rural Ethiopia, 2008 (n = 1994)

Characteristics	Number	Prevalence (95% CI)
Presence of physical violence (n = 1994)		
Never	988	49.5 (47.4, 51.7)
Lifetime occurrence		

Presence of physical violence by onset		
Current (last 12 months)	635	31.8 (29.8, 33.8)
More than 12 months previously	353	17.7 (16.0, 19.4)

Severity of physical violence		
Moderate	270	13.5 (12.0, 15.0)
Severe	718	36.0 (33.9, 38.1)

Physical violence during pregnancy		
No	844	42.3 (40.1, 44.5)
Yes	144	7.2 (1.8, 10.8)

Presence of injury with physical violence		
No	803	39.7 (36.1, 43.3)
Yes	185	8.1 (6.1, 8.3)

Presence of sexual violence		
Lifetime occurrence	1186	59.5 (57.3, 61.6)

Presence of sexual violence by onset		
Current (last 12 months)	975	48.9 (46.7, 51.1)
More than 12 months previously	211	10.6 (9.2, 12.0)

Presence of emotional violence		
Mild (single form)	376	18.9 (17.1, 20.6)
Severe (two or more forms)	177	8.9 (7.6, 10.1)

Sexual violence during childhood (n = 1943)		
Present	166	8.5 (7.3, 9.8)

Spousal control of respondent		
Moderate	1006	50.5 (48.3, 52.7)
High control	99	5.0 (4.0, 6.0)


Any form of intimate partner violence	1435	**72.0 (70.0, 73.9)**


Depressive episode (12-month prevalence)	96	**4.8 (3.9, 5.8)**

### Intimate partner violence and depressive episode

Comparison of the difference in 12-month prevalence of depressive episode by experience of any form of violence by an intimate partner was computed crudely and after adjusting for age, residence, occupational status, poverty status and khat chewing, as presented in Table 4. Depressive episode in the previous 12 months was associated with experience of any form of intimate partner violence, both crudely and after adjustment (Adj. OR = 1.82 95% CI, 1.06, 3.13). Depressive episode was more prevalent among women who had experienced physical violence than women who had never experienced such violence (7.0% compared to 2.7%), with an adjusted odds ratio of OR = 2.56 (95% CI, 1.61, 4.06). Prevalence of depressive episode was significantly higher among women who had experienced physical violence during their last pregnancy (Adj. OR = 4.24; 95% CI, 2.19, 8.21) and physical injury (Adj. OR = 3.59; 95% CI, 1.92, 6.71) compared to those who had never suffered physical violence. Depressive episode was also higher among women for whom the onset of physical violence was more than 12 months previously (Adj. OR = 3.41; 95% CI, 1.99, 5.82) compared to those where violence had started within the last 12 months (Adj. OR = 2.77; 95% CI, 1.69, 4.55).

**Table 4:** Comparison of experience of different forms intimate partner violence with experience of depressive episode in the last 12 months among currently married women in rural Ethiopia, Butajira, 2008 (n = 1994)

Characteristics	Sample	Prevalence No (%)	Crude OR (95% CI)	Adjusted^a^ OR (95% CI)
Physical violence				
Never	1006	27 (2.7)	1.00	1.00
Ever	988	69 (7.0)	2.72 (1.73, 4.29)	2.56 (1.61, 4.06)

Injury during physical violence				
Never violence	1006	27 (2.7)	1.00	1.00
Not Injured	803	50 (6.2)	2.41 (1.49, 3.89)	2.30 (1.41, 3.75)
Injured	185	19 (10.3)	4.15 (2.26, 7.63)	3.59 (1.92, 6.71)

Severity of physical violence				
Never violence	1006	27 (2.7)	1.00	1.00
Moderate	270	17 (6.3)	2.75 (1.50, 5.02)	2.71 (1.47, 4.97)
Severe	718	52 (7.2)	3.13 (1.96, 5.00)	3.09 (1.93, 4.97)

Physical violence. (during last pregnancy)				
Never violence	1006	27 (2.7)	.00	1.00
Not during pregnancy	844	53 (6.3)	2.43 (1.52, 3.90)	2.28 (1.41, 3.70)
Yes, during pregnancy	144	16 (11.1)	4.53 (2.38, 8.64)	4.24 (2.19, 8.21)

Physical violence by onset				
Never	1006	27 (2.7)	1.00	1.00
Current (last 12 months)	635	37 (5.8)	2.70 (1.65, 4.40)	2.77 (1.69, 4.55)
More than 12 months previously	353	32 (9.1)	3.62 (2.13, 6.12)	3.41 (1.99, 5.82)

Presence of sexual violence				
Never	808	36 (4.5)	1.00	1.00
Ever	1186	60 (5.1)	1.14 (0.75, 1.75)	1.14 (0.74, 1.75)

Childhood sexual abuse (n = 1943)				
No	1777	78 (4.4)	1.00	1.00
Yes	166	16 (9.6)	2.32 (1.32, 4.08)	2.00 (1.13, 3.56)

Emotional violence				
Never	1441	42 (2.9)	1.00	1.00
Mild (single form)	376	34 (9.0)	3.32 (2.08, 5.30)	3.19 (1.98, 5.14)
Severe (two/more forms)	177	20 (11.3)	4.24 (2.43, 7.41)	3.90 (2.20, 6.93)

Any form of intim. part. violence				
Never	559	17 (3.0)	1.00	1.00
Present	1435	79 (5.5)	1.86 (1.10, 3.17)	1.82 (1.06, 3.13)

Spousal control of respondent				
Not controlled	889	32 (3.6)	1.00	1.00
Moderate	1006	53 (5.3)	1.49 (0.95, 2.33)	1.52 (0.96, 2.40)
Totally controlled	99	11 (11.1)	3.34 (1.63, 6.87)	3.30 (1.58, 6.90)

Adjusted^a ^: adjusted for age, residency, occupation, poverty status and khat **chewing**

Women who had experienced a depressive episode in the last 12 months were more likely to report experience of sexual violence as a child than women who were not depressed. However, there was no association between depressive episode and sexual violence by an intimate partner (Table 4).

Women with depression reported severe or moderate forms of emotional violence to a higher extent than those without depression. The prevalence of depressive episode was significantly higher among women who reported being totally or moderately controlled by their spouses compared to those who reported not being controlled (Table 4).

## Discussion

### Prevalence of Depressive episode

The 12-month prevalence of depressive episode of 4.8% among currently married women in the present study was relatively low compared to studies from the high income countries, such as the study in six European countries with a six-month prevalence of 17% [36] and a lifetime prevalence of major depression of 13.3% in the Cagliari district of Sardinia [37]. However, the prevalence of depression in the current study is similar to findings from a survey conducted in Australia with a 12-month prevalence of depression of 5.8% [38], as well as a 30-month prevalence of 3.5% [39]. It is also comparable to a study conducted using CIDI in 1996 in Butajira, the same location as the current study, where the lifetime and one-month prevalences of depression among the female population were 4.5% and 3.8% respectively [8]. The relatively low prevalence of depression in this study site may have several explanations, one of which may be the existence of high social capital. It may also be influenced by the instrument used to ascertain depressive episode, with terminologies relating to symptoms of mental illness having different social and cultural contextual meanings [40].

### Prevalence of intimate partner violence

The results of our study indicate that nearly one half of the women surveyed had experienced physical violence at least once in their marital life. Compared to other studies that were part of the multi-country study using a similar instrument, the women in Butajira were more subjected to violence. This could be due both to a lack of policies and legislation relating to intimate partner violence and to cultural norms. Our interview technique was carefully geared to include culturally sensitive terminologies based on focus group discussion with local women, which might have contributed to better recording of intimate partner violence. However, our prevalence result is comparable to an earlier study conducted in the same area in 1996, which found a lifetime prevalence of physical violence by intimate partner of 45% [29], and a Nicaraguan study where 52% reported similar experiences [23].

### Intimate partner violence and depression

Our result of an association between physical violence and depressive episode is consistent with findings from several studies in the high income countries. For example, a study in Australia among 1257 female primary care patients reported that depressed women were significantly more likely to have experienced severe forms of abuse than women who were not depressed [41]. Similarly, a study in a Chinese community in the USA showed a strong association between intimate partner violence and postnatal blues/depression [42].

There are several possible explanations. Firstly, violence by an intimate partner is likely to decrease self-esteem and underpin a negative view of self, the world and the future. Secondly, in Ethiopia, women who experience physical violence are usually humiliated in front of their neighbours, friends and relatives [29], resulting in feelings of shame and withdrawal behaviour, which might further increase isolation and stigmatization [23]. In addition, emotional violence is a form of violence that directly erodes a person's self-esteem, especially intimidation in front of other people. Violence is known to result in recurrent fear and learned helplessness leading to impairment of problem-solving ability. Physical violence combined with emotional trauma can result in feelings of frustration, motivational impairment including passivity, and low self-esteem may lead to depression [21]. In this study, physical violence that begin a year prior to the date of interview had a higher probability of depressive episode than violence that begin within a year of the interview; this finding could be a clue to the probability of depression increasing in parallel with time or with frequency of violence.

Our finding of an association between experiencing sexual violence as a child and depression is consistent with previous studies [43]. Childhood sexual violence is well documented as a risk factor for depression and other mental illnesses in adult life. However, our study failed to show an association between experience of sexual violence by an intimate partner and depressive episode. This might be explained by its hidden nature: it does not take place in public, making the women less stigmatized in the eyes of neighbours and others. The lack of association may also be due to the contextual meaning of sexual violence by an intimate partner, which may not be deemed as violence in a community where men are considered to have the right to have sex with their wives regardless of the woman's consent. This was confirmed by a qualitative study conducted in northwest Ethiopia by Tegbar and co-workers in 2006, which showed that a woman is considered to experience sexual violence only if she is sexually violated by someone other than her spouse (personal communication).

### Spousal control and depressive episode

In this study, reporting being controlled by their spouse was associated with depressive episode in women. This is in accordance with other studies, suggesting that a lack of decisional control among women might be a factor in leading to depression to be more prevalent among women compared to men [5]. Similar to physical or emotional violence, women controlled by their spouses may suffer from feelings of frustration, motivational impairment including passivity and low self-esteem, which could result to adverse mental health consequences including depression [21].

### Limitations and strengths of the study

Our study is limited first by its cross-sectional design with difficulties proving a temporal relationship between explanatory and outcome variables. Another limitation might be recall bias when lifetime experience is included. To minimize this limitation, we used 12-month prevalences of the dependent variables. Even though the research group made great efforts in cultural adaption of the diagnostic instrument CIDI [31], we cannot exclude that CIDI performs less well in this Ethiopian setting. Other possible shortcomings include absence of HIV status, and lack of recording of other hassles of life events which may confound a relationship between intimate partner violence and depressive episode. However, the strengths of our study include minimizing bias by its community-based design utilizing a high-quality study base and a high response rate of 94.6%, one of the highest of the countries participating in the multi-country study [24]. Meticulous training, regular daily supervision of the data collectors, and checking completeness and accuracy are further strengths.

## Conclusion

These findings show that physical violence by an intimate partner including emotional violence and spousal control of women are associated with depressive episode among women in rural Ethiopia. This study expands previous knowledge on partner violence and depression by showing intimate partner violence to be associated with depressive episode in a representative community sample within a low income rural community in a low-income country. Moreover, the study offers a clue that the onset time of violence or a higher frequency of violence is associated with depressive episode. Further research using a longitudinal design to prove such a hypothesis is necessary.

## Competing interests

The authors declare that they have no competing interests.

## Authors' contributions

All authors participated in the design of the study and GK, ME, YB and UH wrote the research protocol. Authors ND, YB, ME and ME participated in the management of data collection, while all authors worked on the statistical analysis. All authors contributed to writing the draft of the manuscript, and have approved the final text.
